# Crystal structure of 5-chloro-2,7-dimethyl-3-[(4-methyl­phenyl)­sulfon­yl]-1-benzo­furan

**DOI:** 10.1107/S1600536814018339

**Published:** 2014-08-16

**Authors:** Hong Dae Choi, Uk Lee

**Affiliations:** a Department of Chemistry, Dongeui University, San 24 Kaya-dong, Busanjin-gu, Busan 614-714, Republic of Korea; b Department of Chemistry, Pukyong National University, 599-1 Daeyeon 3-dong, Nam-gu, Busan 608-737, Republic of Korea

**Keywords:** crystal structure, benzo­furan, 4-methyl­phen­yl, C—H⋯O hydrogen bonds, π–π inter­actions

## Abstract

In the title compound, C_17_H_15_ClO_3_S, the dihedral angle between the planes of the benzo­furan ring system [r.m.s. deviation = 0.008 Å] and the 4-methyl­phenyl ring is 77.29 (4)°. In the crystal, mol­ecules are linked by π–π inter­actions between the benzene rings of neighbouring mol­ecules [centroid–centroid distance = 3.847 (2) Å] and between the benzene and furan rings of neighbouring mol­ecules [centroid–centroid distance = 3.743 (2) Å]. The mol­ecules are stacked along the *a*-axis direction. In addition, pairs of C—H⋯O hydrogen bonds are observed between inversion-related dimers: these generate *R*_2_^2^(12) loops.

## Related literature   

For the pharmaceutical properties of benzo­furan compounds, see: Aslam *et al.* (2009 ▶); Galal *et al.* (2009 ▶); Howlett *et al.* (1999 ▶); Khan *et al.* (2005 ▶); Ono *et al.* (2002 ▶). For natural products with a benzo­furan ring, see: Akgul & Anil (2003 ▶); Soekamto *et al.* (2003 ▶). For the synthesis of the starting material 5-chloro-2,7-dimethyl-3-(4-methyl­phenyl­sulfan­yl)-1-benzo­furan, see: Choi *et al.* (1999 ▶). For a related structure, see: Choi *et al.* (2014 ▶).
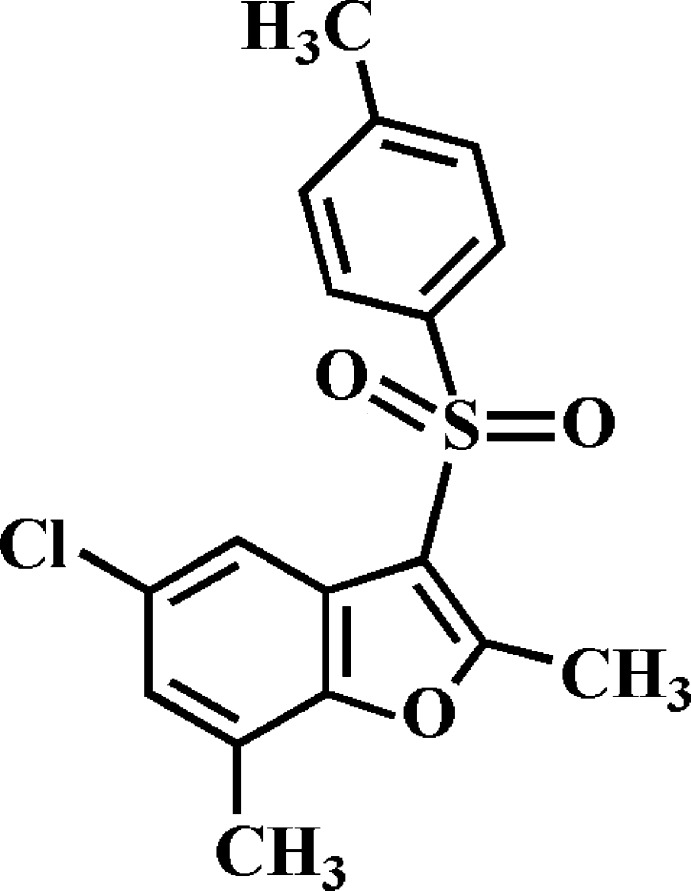


## Experimental   

### Crystal data  

C_17_H_15_ClO_3_S
*M* *_r_* = 334.80
Triclinic, 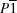

*a* = 8.2757 (2) Å
*b* = 9.6740 (2) Å
*c* = 10.1564 (2) Å
α = 76.655 (1)°
β = 75.673 (1)°
γ = 76.355 (1)°
*V* = 752.64 (3) Å^3^
*Z* = 2
Mo *K*α radiation
μ = 0.40 mm^−1^
*T* = 173 K
0.35 × 0.32 × 0.25 mm

### Data collection  

Bruker SMART APEXII CCD diffractometer
Absorption correction: multi-scan (*SADABS*; Bruker, 2009 ▶) *T*_min_ = 0.871, *T*_max_ = 0.905
14105 measured reflections
3745 independent reflections
3274 reflections with *I* > 2σ(*I*)
*R*_int_ = 0.023

### Refinement  

*R*[*F* ^2^ > 2σ(*F* ^2^)] = 0.035
*wR*(*F* ^2^) = 0.099
*S* = 1.05
3745 reflections
202 parameters
H-atom parameters constrained
Δρ_max_ = 0.34 e Å^−3^
Δρ_min_ = −0.32 e Å^−3^

### 

Data collection: *APEX2* (Bruker, 2009 ▶); cell refinement: *SAINT* (Bruker, 2009 ▶); data reduction: *SAINT*; program(s) used to solve structure: *SHELXS97* (Sheldrick, 2008 ▶); program(s) used to refine structure: *SHELXL97* (Sheldrick, 2008 ▶); molecular graphics: *ORTEP-3 for Windows* (Farrugia, 2012 ▶) and *DIAMOND* (Brandenburg, 1998 ▶; software used to prepare material for publication: *SHELXL97*.

## Supplementary Material

Crystal structure: contains datablock(s) I. DOI: 10.1107/S1600536814018339/hb7272sup1.cif

Structure factors: contains datablock(s) I. DOI: 10.1107/S1600536814018339/hb7272Isup2.hkl

Supporting information file. DOI: 10.1107/S1600536814018339/hb7272Isup3.cml

**Fig. 1.**
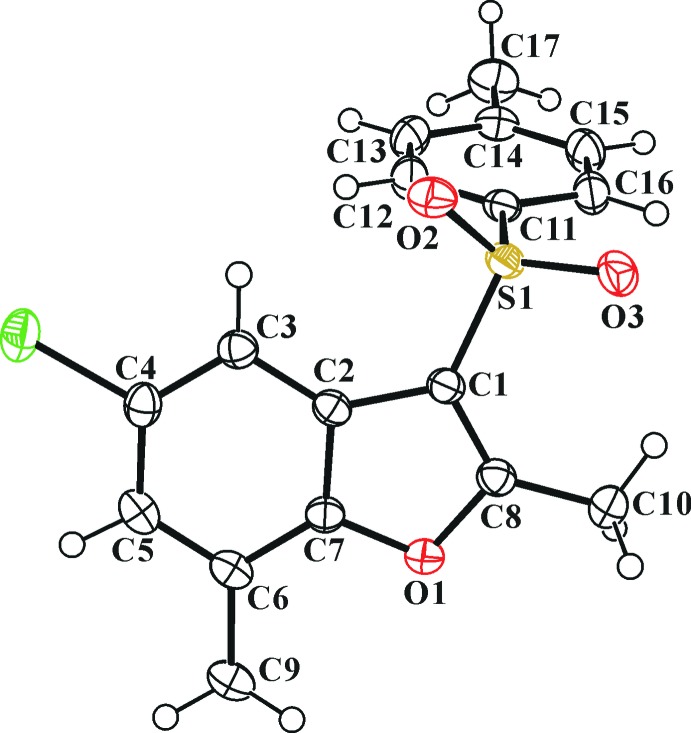
The molecular structure of the title compound with displacement ellipsoids drawn at the 50% probability level.

**Fig. 2.**
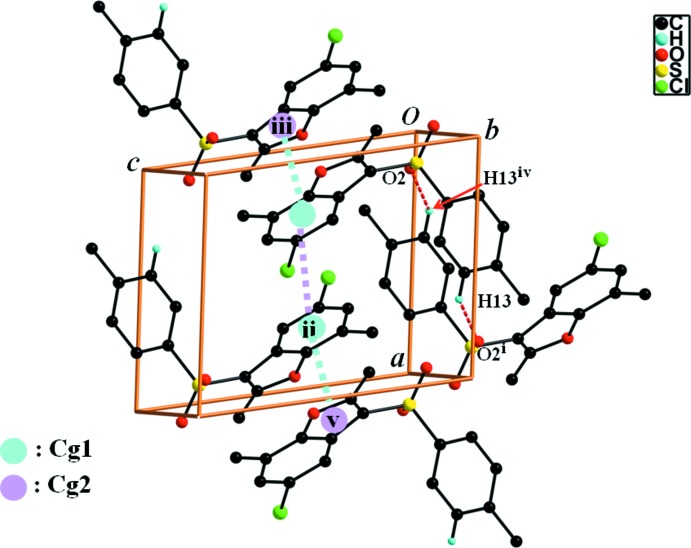
A view of the C—H···O and π···π interactions (dotted lines) in the crystal structure of the title compound. H atoms non-participating in hydrogen-bonding were omitted for clarity. [Symmetry codes: (i) - x + 1, - y + 1, - z; (ii) - x + 1, - y + 1, - z + 1; (iii) - x, - y + 1, - z + 1; (iv) x + 1, y, z; (v) x + 1 , y, z.]

. DOI: 10.1107/S1600536814018339/hb7272fig1.tif
The mol­ecular structure of the title compound with displacement ellipsoids drawn at the 50% probability level.

x y z x y z x y z x y z x y z . DOI: 10.1107/S1600536814018339/hb7272fig2.tif
A view of the C—H⋯O and π⋯π inter­actions (dotted lines) in the crystal structure of the title compound. H atoms non-participating in hydrogen-bonding were omitted for clarity. [Symmetry codes: (i) − *x* + 1, − *y* + 1, − *z*; (ii) − *x* + 1, − *y* + 1, − *z* + 1; (iii) − *x*, − *y* + 1, − *z* + 1; (iv) *x* + 1, *y*, *z*; (v) *x* + 1, *y*, *z*.]

CCDC reference: 1018954

Additional supporting information:  crystallographic information; 3D view; checkCIF report

## Figures and Tables

**Table 1:** Hydrogen-bond geometry (Å, °)

*D*—H⋯*A*	*D*—H	H⋯*A*	*D*⋯*A*	*D*—H⋯*A*
C13—H13⋯O2^i^	0.95	2.52	3.269 (2)	136

Symmetry code: (i) 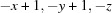.

## supplementary crystallographic information

### S1. Comment

Benzofuran compounds show various pharmacological properties such as antibacterial and antifungal, antitumor and antiviral, antimicrobial activities (Aslam *et al.* 2009, Galal *et al.*, 2009, Khan *et al.*, 2005), and potential inhibitor of β-amyloid aggregation (Howlett *et al.*, 1999, Ono *et al.*, 2002). These benzofuran compounds are widely occurring in nature (Akgul & Anil, 2003, Soekamto *et al.*, 2003). As a part of our ongoing project of 3-arylsulfonyl-5-chloro-2,7-dimethyl-1-benzofuran derivatives containing 3-methylphenylsulfonyl substituent in 3-position (Choi *et al.*, 2014), we report herein on the crystal structure of the title compound.

In the title molecule (Fig. 1), the benzofuran unit is essentially planar, with a mean deviation of 0.008 (1) Å from the least-squares plane defined by the nine constituent atoms. The 4-fluorophenyl ring is essentially planar, with a mean deviation of 0.006 (1) Å from the least-squares plane defined by the six constituent atoms. The dihedral angle formed by the benzofuran ring system and the 4-methylphenyl ring is 77.29 (4)°. In the crystal structure (Fig. 2), molecules are linked by π···π interactions between the benzene rings of neighbouring molecules with a Cg1···Cg1^ii^ distance of 3.847 (2) Å and an interplanar distance of 3.479 (2) Å resulting in a slippage of 1.642 (2) Å (Cg1 is the centroid of the C2–C7 benzene ring), and between the benzene and furan rings of neighbouring molecules with a Cg1···Cg2^iii^ distance of 3.743 (2) Å and an interplanar distance of 3.595 (2) Å resulting in a slippage of 1.042 (2) Å (Cg2 is the centroid of the C1/C2/C7/O1/C8 furan ring). The molecules are stacked along the *a*-axis direction. In addition, intermolecular C—H···O hydrogen bonds (Table 1) are observed between inversion-related dimers.

### S2. Experimental

The starting material 5-chloro-2,7-dimethyl-3-(4-methylphenylsulfanyl)-1-benzofuran was prepared by literature method (Choi *et al.* 1999). 3-Chloroperoxybenzoic acid (77%, 515 mg, 2.3 mmol) was added in small portions to a stirred solution of 5-chloro-3-(4-methylphenylsulfanyl)-2,7-dimethyl-1-benzofuran (333 mg, 1.1 mmol) in dichloromethane (30 ml) at 273 K. After being stirred at room temperature for 8h, the mixture was washed with saturated sodium bicarbonate solution (2 × 20 ml) and the organic layer was separated, dried over magnesium sulfate, filtered and concentrated at reduced pressure. The residue was purified by column chromatography (hexane-ethyl acetate, 4:1 *v*/*v*) to afford the title compound as a colorless solid [yield 69% (254 mg); m.p. 468–469 K; *R*_f_ = 0.61 (hexane–ethyl acetate, 4:1 *v*/*v*)]. Colourless blocks were prepared by slow evaporation of a solution of the title compound (25 mg) in ethyl acetate (20 ml) at room temperature.

### S3. Refinement

All H atoms were positioned geometrically and refined using a riding model, with C—H = 0.95 Å for aryl and 0.98 Å for methyl H atoms, *U*_iso_ (H) = 1.2*U*_eq_ (C) for aryl and 1.5*U*_eq_ (C) for methyl H atoms.The positions of methyl hydrogens were optimized using the SHELXL-97's command AFIX 137 (Sheldrick, 2008).

### Crystal data

**Table:**

C_17_H_15_ClO_3_S	*Z* = 2
*M**_r_* = 334.80	*F*(000) = 348
Triclinic, *P*1	*D*_x_ = 1.477 Mg m^−^^3^
Hall symbol: -P 1	Melting point = 469–468 K
*a* = 8.2757 (2) Å	Mo *K*α radiation, λ = 0.71073 Å
*b* = 9.6740 (2) Å	Cell parameters from 5796 reflections
*c* = 10.1564 (2) Å	θ = 2.6–28.4°
α = 76.655 (1)°	µ = 0.40 mm^−^^1^
β = 75.673 (1)°	*T* = 173 K
γ = 76.355 (1)°	Block, colourless
*V* = 752.64 (3) Å^3^	0.35 × 0.32 × 0.25 mm

### Data collection

**Table:**

Bruker SMART APEXII CCD diffractometer	3745 independent reflections
Radiation source: rotating anode	3274 reflections with *I* > 2σ(*I*)
Graphite multilayer monochromator	*R*_int_ = 0.023
Detector resolution: 10.0 pixels mm^-1^	θ_max_ = 28.4°, θ_min_ = 2.1°
φ and ω scans	*h* = −11→10
Absorption correction: multi-scan (*SADABS*; Bruker, 2009)	*k* = −12→12
*T*_min_ = 0.871, *T*_max_ = 0.905	*l* = −13→13
14105 measured reflections	

### Refinement

**Table:**

Refinement on *F*^2^	Primary atom site location: structure-invariant direct methods
Least-squares matrix: full	Secondary atom site location: difference Fourier map
*R*[*F*^2^ > 2σ(*F*^2^)] = 0.035	Hydrogen site location: difference Fourier map
*wR*(*F*^2^) = 0.099	H-atom parameters constrained
*S* = 1.05	*w* = 1/[σ^2^(*F*_o_^2^) + (0.050*P*)^2^ + 0.3424*P*] where *P* = (*F*_o_^2^ + 2*F*_c_^2^)/3
3745 reflections	(Δ/σ)_max_ < 0.001
202 parameters	Δρ_max_ = 0.34 e Å^−^^3^
0 restraints	Δρ_min_ = −0.32 e Å^−^^3^

### Special details

**Table:**

Experimental. ^1^H NMR (δ p.p.m., CDCl3, 400 Hz): 7.87 (d, J = 8.56 Hz, 2H), 7.68 (s, 1H),7.31 (d, J = 8.24 Hz, 2H), 7.07-7.09 (m, 1H), 2.80 (s, 3H), 2.42 (s, 3H), 2.39 (s, 3H).
Geometry. All esds (except the esd in the dihedral angle between two l.s. planes) are estimated using the full covariance matrix. The cell esds are taken into account individually in the estimation of esds in distances, angles and torsion angles; correlations between esds in cell parameters are only used when they are defined by crystal symmetry. An approximate (isotropic) treatment of cell esds is used for estimating esds involving l.s. planes.
Refinement. Refinement of F^2^ against ALL reflections. The weighted R-factor wR and goodness of fit S are based on F^2^, conventional R-factors R are based on F, with F set to zero for negative F^2^. The threshold expression of F^2^ > 2sigma(F^2^) is used only for calculating R-factors(gt) etc. and is not relevant to the choice of reflections for refinement. R-factors based on F^2^ are statistically about twice as large as those based on F, and R- factors based on ALL data will be even larger.

### Fractional atomic coordinates and isotropic or equivalent isotropic displacement parameters (Å^2^)

**Table:**

	*x*	*y*	*z*	*U*_iso_*/*U*_eq_	
Cl1	0.49131 (6)	0.18481 (4)	0.50226 (5)	0.03579 (12)	
S1	0.09180 (5)	0.67869 (4)	0.15275 (4)	0.02306 (11)	
O1	0.06794 (14)	0.75353 (12)	0.52188 (11)	0.0243 (2)	
O2	0.11863 (15)	0.52962 (13)	0.14105 (12)	0.0301 (3)	
O3	−0.05754 (14)	0.77699 (13)	0.11729 (12)	0.0312 (3)	
C1	0.09965 (19)	0.67929 (16)	0.32240 (14)	0.0219 (3)	
C2	0.19361 (19)	0.56512 (16)	0.40987 (14)	0.0216 (3)	
C3	0.29224 (19)	0.42829 (16)	0.39832 (15)	0.0240 (3)	
H3	0.3099	0.3878	0.3179	0.029*	
C4	0.3628 (2)	0.35505 (17)	0.51112 (16)	0.0259 (3)	
C5	0.3387 (2)	0.41057 (18)	0.63123 (16)	0.0276 (3)	
H5	0.3907	0.3547	0.7051	0.033*	
C6	0.2401 (2)	0.54586 (18)	0.64457 (15)	0.0252 (3)	
C7	0.16990 (19)	0.61807 (16)	0.53085 (15)	0.0224 (3)	
C8	0.02745 (19)	0.78847 (17)	0.39395 (15)	0.0238 (3)	
C9	0.2140 (2)	0.6124 (2)	0.76979 (16)	0.0324 (4)	
H9A	0.3006	0.6710	0.7560	0.049*	
H9B	0.2235	0.5356	0.8511	0.049*	
H9C	0.1010	0.6738	0.7841	0.049*	
C10	−0.0811 (2)	0.93214 (18)	0.36327 (17)	0.0302 (3)	
H10A	−0.1029	0.9475	0.2699	0.045*	
H10B	−0.0233	1.0074	0.3691	0.045*	
H10C	−0.1891	0.9368	0.4305	0.045*	
C11	0.27059 (19)	0.74903 (17)	0.05186 (14)	0.0230 (3)	
C12	0.4317 (2)	0.66660 (18)	0.05643 (17)	0.0299 (3)	
H12	0.4454	0.5743	0.1148	0.036*	
C13	0.5723 (2)	0.72047 (19)	−0.02508 (18)	0.0325 (4)	
H13	0.6829	0.6650	−0.0214	0.039*	
C14	0.5538 (2)	0.85464 (19)	−0.11220 (16)	0.0294 (3)	
C15	0.3924 (2)	0.93542 (19)	−0.11401 (17)	0.0339 (4)	
H15	0.3785	1.0278	−0.1723	0.041*	
C16	0.2502 (2)	0.88400 (18)	−0.03225 (17)	0.0300 (3)	
H16	0.1399	0.9409	−0.0339	0.036*	
C17	0.7084 (2)	0.9091 (2)	−0.2026 (2)	0.0413 (4)	
H17A	0.7429	0.8668	−0.2865	0.062*	
H17B	0.8014	0.8811	−0.1522	0.062*	
H17C	0.6815	1.0148	−0.2279	0.062*	

### Atomic displacement parameters (Å^2^)

**Table:**

	*U*^11^	*U*^22^	*U*^33^	*U*^12^	*U*^13^	*U*^23^
Cl1	0.0358 (2)	0.0238 (2)	0.0425 (2)	−0.00058 (16)	−0.00815 (18)	−0.00056 (16)
S1	0.02253 (19)	0.0282 (2)	0.01904 (17)	−0.00402 (14)	−0.00553 (13)	−0.00488 (13)
O1	0.0263 (5)	0.0257 (5)	0.0213 (5)	−0.0033 (4)	−0.0049 (4)	−0.0068 (4)
O2	0.0349 (6)	0.0318 (6)	0.0270 (6)	−0.0095 (5)	−0.0057 (5)	−0.0093 (5)
O3	0.0249 (6)	0.0400 (7)	0.0274 (6)	−0.0017 (5)	−0.0096 (5)	−0.0038 (5)
C1	0.0221 (7)	0.0248 (7)	0.0190 (6)	−0.0043 (6)	−0.0042 (5)	−0.0043 (5)
C2	0.0217 (7)	0.0238 (7)	0.0194 (6)	−0.0066 (6)	−0.0034 (5)	−0.0027 (5)
C3	0.0247 (7)	0.0241 (7)	0.0231 (7)	−0.0058 (6)	−0.0035 (6)	−0.0043 (6)
C4	0.0241 (7)	0.0220 (7)	0.0291 (7)	−0.0047 (6)	−0.0046 (6)	−0.0001 (6)
C5	0.0288 (8)	0.0304 (8)	0.0241 (7)	−0.0099 (6)	−0.0087 (6)	0.0019 (6)
C6	0.0261 (8)	0.0307 (8)	0.0204 (7)	−0.0106 (6)	−0.0047 (6)	−0.0026 (6)
C7	0.0220 (7)	0.0238 (7)	0.0213 (7)	−0.0056 (6)	−0.0028 (5)	−0.0045 (5)
C8	0.0227 (7)	0.0268 (8)	0.0218 (7)	−0.0054 (6)	−0.0038 (5)	−0.0043 (6)
C9	0.0376 (9)	0.0400 (10)	0.0227 (7)	−0.0106 (7)	−0.0086 (6)	−0.0061 (7)
C10	0.0296 (8)	0.0278 (8)	0.0318 (8)	0.0006 (6)	−0.0078 (6)	−0.0073 (6)
C11	0.0243 (7)	0.0269 (8)	0.0176 (6)	−0.0042 (6)	−0.0035 (5)	−0.0054 (5)
C12	0.0282 (8)	0.0270 (8)	0.0309 (8)	−0.0019 (6)	−0.0062 (6)	−0.0011 (6)
C13	0.0230 (8)	0.0355 (9)	0.0353 (9)	−0.0001 (7)	−0.0034 (6)	−0.0074 (7)
C14	0.0305 (8)	0.0351 (9)	0.0231 (7)	−0.0090 (7)	−0.0013 (6)	−0.0082 (6)
C15	0.0366 (9)	0.0313 (9)	0.0286 (8)	−0.0061 (7)	−0.0058 (7)	0.0033 (7)
C16	0.0271 (8)	0.0309 (8)	0.0277 (8)	−0.0008 (6)	−0.0067 (6)	−0.0005 (6)
C17	0.0350 (10)	0.0507 (12)	0.0362 (9)	−0.0159 (9)	0.0027 (7)	−0.0069 (8)

### Geometric parameters (Å, º)

**Table:**

Cl1—C4	1.7452 (16)	C9—H9B	0.9800
S1—O2	1.4346 (12)	C9—H9C	0.9800
S1—O3	1.4384 (11)	C10—H10A	0.9800
S1—C1	1.7417 (14)	C10—H10B	0.9800
S1—C11	1.7621 (16)	C10—H10C	0.9800
O1—C8	1.3690 (17)	C11—C16	1.384 (2)
O1—C7	1.3779 (18)	C11—C12	1.388 (2)
C1—C8	1.357 (2)	C12—C13	1.386 (2)
C1—C2	1.448 (2)	C12—H12	0.9500
C2—C7	1.391 (2)	C13—C14	1.391 (2)
C2—C3	1.395 (2)	C13—H13	0.9500
C3—C4	1.385 (2)	C14—C15	1.382 (2)
C3—H3	0.9500	C14—C17	1.507 (2)
C4—C5	1.396 (2)	C15—C16	1.387 (2)
C5—C6	1.386 (2)	C15—H15	0.9500
C5—H5	0.9500	C16—H16	0.9500
C6—C7	1.388 (2)	C17—H17A	0.9800
C6—C9	1.501 (2)	C17—H17B	0.9800
C8—C10	1.478 (2)	C17—H17C	0.9800
C9—H9A	0.9800		
			
O2—S1—O3	119.70 (7)	C6—C9—H9C	109.5
O2—S1—C1	106.30 (7)	H9A—C9—H9C	109.5
O3—S1—C1	109.53 (7)	H9B—C9—H9C	109.5
O2—S1—C11	107.88 (7)	C8—C10—H10A	109.5
O3—S1—C11	107.90 (7)	C8—C10—H10B	109.5
C1—S1—C11	104.52 (7)	H10A—C10—H10B	109.5
C8—O1—C7	107.01 (11)	C8—C10—H10C	109.5
C8—C1—C2	107.66 (13)	H10A—C10—H10C	109.5
C8—C1—S1	126.70 (12)	H10B—C10—H10C	109.5
C2—C1—S1	125.56 (12)	C16—C11—C12	120.52 (15)
C7—C2—C3	119.58 (13)	C16—C11—S1	120.29 (12)
C7—C2—C1	104.47 (13)	C12—C11—S1	119.18 (12)
C3—C2—C1	135.95 (14)	C13—C12—C11	119.26 (15)
C4—C3—C2	115.94 (14)	C13—C12—H12	120.4
C4—C3—H3	122.0	C11—C12—H12	120.4
C2—C3—H3	122.0	C12—C13—C14	120.91 (15)
C3—C4—C5	123.62 (15)	C12—C13—H13	119.5
C3—C4—Cl1	118.36 (13)	C14—C13—H13	119.5
C5—C4—Cl1	118.01 (12)	C15—C14—C13	118.81 (15)
C6—C5—C4	121.09 (14)	C15—C14—C17	121.28 (16)
C6—C5—H5	119.5	C13—C14—C17	119.92 (16)
C4—C5—H5	119.5	C14—C15—C16	121.12 (15)
C5—C6—C7	114.72 (14)	C14—C15—H15	119.4
C5—C6—C9	123.22 (14)	C16—C15—H15	119.4
C7—C6—C9	122.03 (15)	C11—C16—C15	119.36 (15)
O1—C7—C6	124.46 (14)	C11—C16—H16	120.3
O1—C7—C2	110.50 (13)	C15—C16—H16	120.3
C6—C7—C2	125.05 (15)	C14—C17—H17A	109.5
C1—C8—O1	110.35 (13)	C14—C17—H17B	109.5
C1—C8—C10	134.26 (14)	H17A—C17—H17B	109.5
O1—C8—C10	115.39 (13)	C14—C17—H17C	109.5
C6—C9—H9A	109.5	H17A—C17—H17C	109.5
C6—C9—H9B	109.5	H17B—C17—H17C	109.5
H9A—C9—H9B	109.5		
			
O2—S1—C1—C8	−156.13 (14)	C1—C2—C7—O1	0.67 (16)
O3—S1—C1—C8	−25.47 (16)	C3—C2—C7—C6	1.2 (2)
C11—S1—C1—C8	89.91 (15)	C1—C2—C7—C6	−178.72 (14)
O2—S1—C1—C2	27.64 (15)	C2—C1—C8—O1	0.08 (17)
O3—S1—C1—C2	158.29 (13)	S1—C1—C8—O1	−176.71 (11)
C11—S1—C1—C2	−86.32 (14)	C2—C1—C8—C10	179.75 (17)
C8—C1—C2—C7	−0.45 (17)	S1—C1—C8—C10	3.0 (3)
S1—C1—C2—C7	176.38 (11)	C7—O1—C8—C1	0.34 (17)
C8—C1—C2—C3	179.68 (17)	C7—O1—C8—C10	−179.40 (13)
S1—C1—C2—C3	−3.5 (3)	O2—S1—C11—C16	136.28 (13)
C7—C2—C3—C4	−1.0 (2)	O3—S1—C11—C16	5.64 (15)
C1—C2—C3—C4	178.86 (16)	C1—S1—C11—C16	−110.88 (14)
C2—C3—C4—C5	0.5 (2)	O2—S1—C11—C12	−42.86 (14)
C2—C3—C4—Cl1	−178.70 (11)	O3—S1—C11—C12	−173.49 (13)
C3—C4—C5—C6	0.0 (2)	C1—S1—C11—C12	69.99 (14)
Cl1—C4—C5—C6	179.14 (12)	C16—C11—C12—C13	−0.5 (2)
C4—C5—C6—C7	0.1 (2)	S1—C11—C12—C13	178.65 (13)
C4—C5—C6—C9	−178.22 (15)	C11—C12—C13—C14	−0.8 (3)
C8—O1—C7—C6	178.75 (14)	C12—C13—C14—C15	1.5 (3)
C8—O1—C7—C2	−0.64 (16)	C12—C13—C14—C17	−178.38 (17)
C5—C6—C7—O1	180.00 (14)	C13—C14—C15—C16	−0.8 (3)
C9—C6—C7—O1	−1.6 (2)	C17—C14—C15—C16	179.00 (17)
C5—C6—C7—C2	−0.7 (2)	C12—C11—C16—C15	1.1 (3)
C9—C6—C7—C2	177.66 (15)	S1—C11—C16—C15	−178.02 (13)
C3—C2—C7—O1	−179.44 (13)	C14—C15—C16—C11	−0.4 (3)

### Hydrogen-bond geometry (Å, º)

**Table:**

*D*—H···*A*	*D*—H	H···*A*	*D*···*A*	*D*—H···*A*
C13—H13···O2^i^	0.95	2.52	3.269 (2)	136

Symmetry code: (i) −*x*+1, −*y*+1, −*z*.
