# Supplementary figures and images for: Crystal structure of 5-chloro-2,7-dimethyl-3-[(4-methyl­phenyl)­sulfon­yl]-1-benzo­furan
Source: Acta Crystallogr Sect E Struct Rep Online. 2014 Aug 16;70(Pt 9):o1018–9. doi: 10.1107/S1600536814018339 (PMC4186162; doi:10.1107/S1600536814018339)

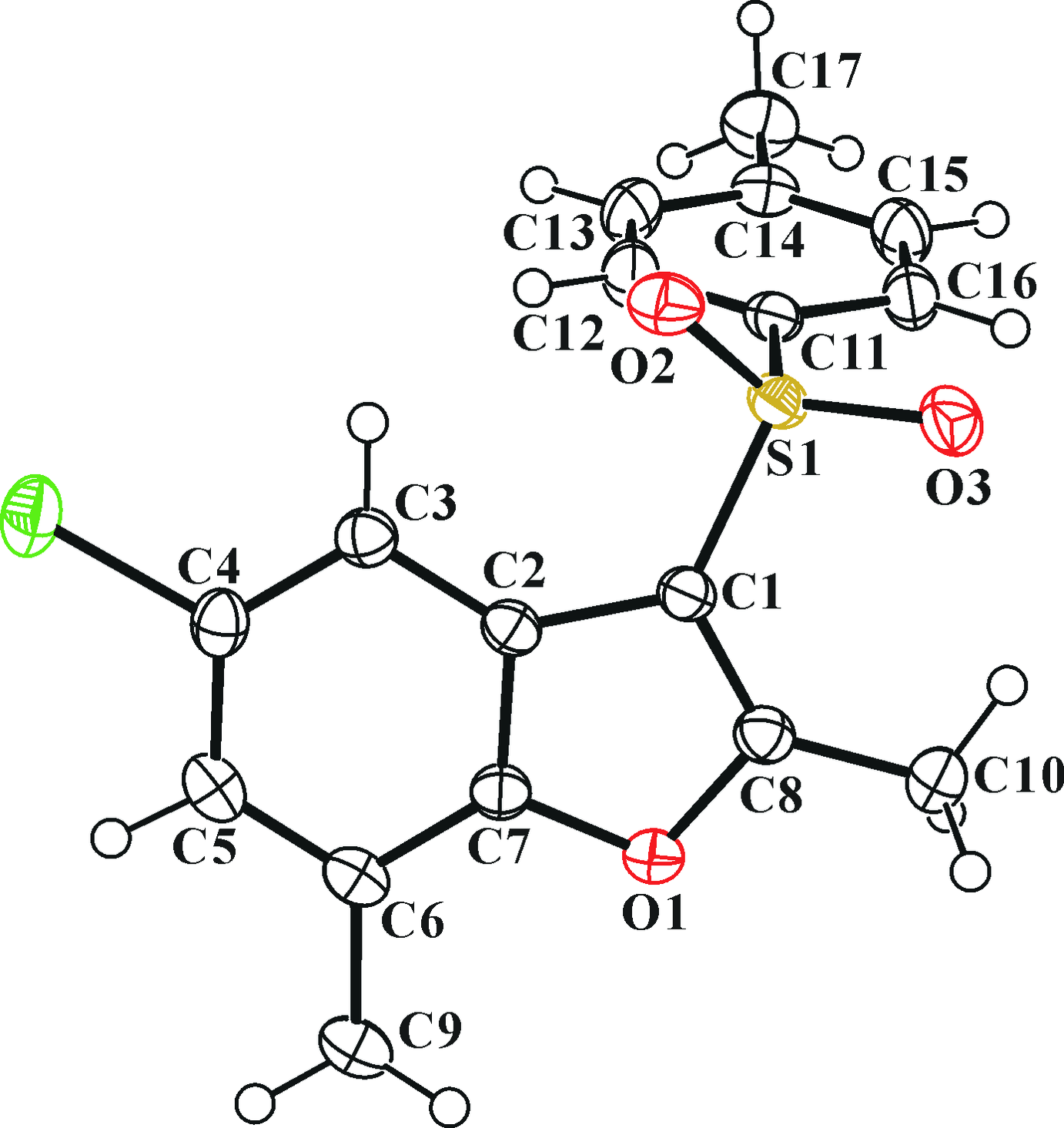

Supplement: Supplementary file 4 [file e-70-o1018-fig1.tif]

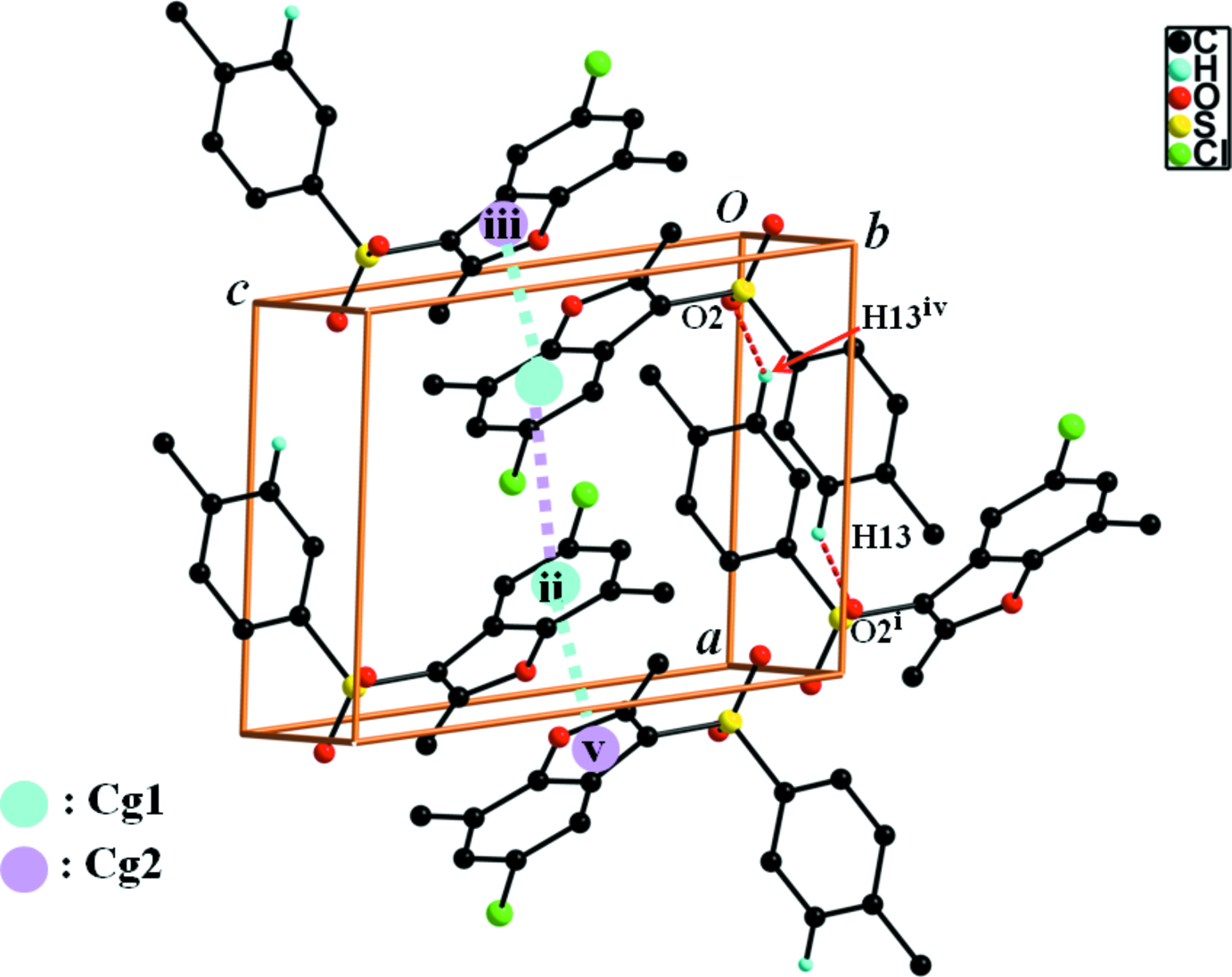

Supplement: Supplementary file 5 [file e-70-o1018-fig2.tif]
